# Regularized win ratio regression for variable selection and risk prediction, with an application to a cardiovascular trial

**DOI:** 10.21203/rs.3.rs-5836301/v1

**Published:** 2025-02-07

**Authors:** Lu Mao

**Affiliations:** Department of Biostatistics and Medical Informatics, School of Medicine and Public Health, University of Wisconsin-Madison, 610 Walnut St, Room 207A , Madison, 53726, WI, USA.

**Keywords:** Concordance index, Hierarchical composite endpoints, Elastic net, Lasso, Proportional win-fractions

## Abstract

**Background:**

The win ratio has been widely used in the analysis of hierarchical composite endpoints, which prioritize critical outcomes such as mortality over nonfatal, secondary events. Although a regression framework exists to incorporate covariates, it is limited to low-dimensional datasets and may struggle with numerous predictors. This gap necessitates a robust variable selection method tailored to the win ratio framework.

**Methods:**

We propose an elastic net-type regularization approach for win ratio regression, extending the proportional win-fractions (PW) model in low-dimensional settings. The method addresses key challenges, including adapting pairwise comparisons to penalized regression, optimizing model selection through subject-level cross-validation, and defining performance metrics via a generalized concordance index. The procedures are implemented in the wrnet R-package, publicly available at https://lmaowisc.github.io/wrnet/.

**Results:**

Simulation studies demonstrate that wrnet outperforms traditional (regularized) Cox regression for time-to-first-event analysis, particularly in scenarios with differing covariate effects on mortality and nonfatal events. When applied to data from the HF-ACTION trial, the method identified prognostic variables and achieved superior predictive accuracy compared to regularized Cox models, as measured by overall and component-specific concordance indices.

**Conclusion:**

The wrnet approach combines the interpretability and clinical relevance of the win ratio with the scalability and robustness of elastic net regularization. The accompanying R-package provides a user-friendly interface for routine application of the procedures, whenever appropriate. Future research could explore additional applications or refine the methodology to address non-proportionalities in win-loss risks and nonlinearities in covariate effects.

## 1 Background

The win ratio has become a powerful statistical tool for analyzing hierarchical composite endpoints [1–4]. It begins by pairing all patients from the treatment to those from the control group. Then, for each pair, a “win” is assigned to the group whose patient fares better based on a user-defined hierarchy of outcomes. Typically, this hierarchy prioritizes death over nonfatal events, such as hospitalization, ensuring that more severe outcomes drive the analysis. If neither patient in the pair demonstrates superiority on the prioritized outcomes (e.g., both survive and experience no or the same number of hospitalizations), the pair is considered a tie. The win ratio is then calculated as the relative proportions of wins to losses, summarizing the treatment effect in a manner that respects the clinical importance of different outcomes.

Originally introduced for two-sample comparisons, the win ratio has been extended to regression, with the proportional win-fractions (PW) model serving as the primary framework [5]. In this setting, the win ratio is modeled as a multiplicative function of covariate differences, allowing a well-rounded assessment of how each patient characteristic contributes to the chance of “winning”. The regression framework is particularly useful in applications such as heart failure trials, where a variety of clinical, functional, or echocardiographic baseline measurements can shape patient outcomes over the follow-up period [6]. With prioritized comparisons, the PW model ensures that all mortality data are not only included but also emphasized in the analysis. By contrast, the traditional Cox model [7] for time to the first event often disproportionately relies on the nonfatal component, as these events typically occur earlier and more frequently than deaths.

However, win ratio regression remains constrained to standard, low-dimensional settings. For the PW model, this means that the number of covariates must be much smaller than the sample size to avoid overfitting. As a result, investigators are often obliged to pre-select a subset of relevant features from a potentially large pool of candidate variables – a task that is both challenging and subjective.

In the landmark HF-ACTION trial, for example, a large number of baseline variables were recorded, including patient demographics, medical history, functional measurements (e.g., six-minute walk test, peak VO2), echocardiographic parameters (e.g., left ventricular ejection fraction), biomarker levels (e.g., NT-proBNP, troponins), and cardiopulominary exercise (CPX) test results ((e.g., ventilatory threshold, oxygen uptake efficiency slope, and heart rate recovery). Manually assessing the relevance of each variable and deciding its inclusion in the model would be impractical and error-prone. Even in cases where the sample size is sufficiently large to accommodate all variables, a more parsimonious model is often preferred for interpretability and usability. Thus, an automated procedure that effectively balances variable selection with model performance would greatly benefit researchers in modeling hierarchical composite endpoints through the win ratio.

For other models, regularization techniques such as the lasso and elastic net have provided satisfactory solutions. The lasso (least absolute shrinkage and selection operator) regularizes regression models by penalizing the absolute values of regression coefficients [8, 9]. Due to the angular contours of the $L_{1}$ norm (sum of the absolute values), the lasso sets coefficients for less important variables to exactly zero, resulting in a sparse solution for the model parameters. However, the lasso may struggle when predictors are highly correlated, as it tends to arbitrarily select one variable from a group of correlated predictors, potentially reducing model stability. To address this limitation, the elastic net was introduced, combining the strengths of the lasso and ridge regression [10]. Its penalty term is a weighted combination of $L_{1}$ and $L_{2}$ penalties, encouraging sparsity while allowing for grouped selection, so that correlated variables are either included or excluded together. The balance between $L_{1}$ and $L_{2}$ penalties is controlled by a user-specified mixing parameter, while the overall penalty controlled by a tuning parameter to be determined in a data-dependent way, such as through cross-validation. This general approach has been applied to linear models, generalized linear models (GLMs) [11], and the Cox model [12].

Adapting the elastic net to the win ratio requires more thinking. First, the standard solution to win ratio regression is not framed as a minimization problem – as is the case for linear models, GLMs, or the Cox model, where solutions are obtained by minimizing the residual sum of squares, negative log-likelihood, or negative log-partial likelihood, respectively. Rather, the win ratio parameter is obtained by solving a pairwise estimating equation that has mean zero under the model. Therefore, before applying an elastic net-type penalty, we need to first define an appropriate objective function to be penalized.

Second, a win ratio model targets the pair rather than the subject. However, if the pair is treated naively as the basic unit of observation, it can have serious consequences on model training, validation and testing, as different pairs used for different purposes may in fact be correlated through shared subjects. This raises another question regarding the appropriate metric to evaluate model performance. While the accuracy of correctly classifying a win or loss work naturally for a set of *pairs*, a more principled approach is needed when considering an independent set of *subjects*.

By addressing these problems, we aim to develop a coherent approach to elastic net-type regularization for the win ratio. Specifically, we re-formulate the standard win ratio solution as a minimizer of an objective function that mimics the negative log-likelihood of a pairwise “conditional” logistic regression for classifying win versus loss among “comparable” pairs. This allows us to use existing software routines as a numerical shortcut to fit regularized win ratio models with given tuning parameters. To identify the optimal tuning, however, we caution against directly applying standard cross-validation procedures designed for logistic regression, as the correlations among the pairs compromise the objectivity of the validation process and bias the results toward overfitting. Instead, we propose cross-validation on folds of partitioned *subjects*, using a generalized concordance index to measure the agreement between a model-fitted risk score and the hierarchical outcomes observed.

The rest of the paper is outlined as follows. The next section reviews the standard PW model and details the methodological framework for fitting, tuning, and testing regularized models. A simple, reproducible code example is provided to demonstrate the use of the wrnet R-package developed to streamline this process. Simulation studies are then presented to evaluate the performance of the proposed methods, especially in comparison with regularized Cox models for time-to-first-event analysis on variable selection and predictive accuracy. As an illustration, we apply the regularized win ratio approach to a real-world dataset from the HF-ACTION trial, highlighting its utility in selecting prognostic variables and building interpretable models. Finally, we conclude the paper with a discussion of the practical implications of the findings, limitations of the current approach, and potential directions for future research.

## 2 Methods

### 2.1 Review of standard PW model

Let D denote the survival time, and define $N_{D}(t)=I(D\leq t)$ as the counting process for death, where I(⋅) is the indicator function. Similarly, let N(t) count the cumulative number of nonfatal events (e.g., hospitalizations) by time t. To simplify notation, denote the complete set of outcomes data available up to time t by Y(t), that is,

$$Y(t)=\left\{N_{D}(u),N(u):0\leq u\leq t\right\}.$$

In the simpler case where N(t)=I(T≤t) for a nonfatal event time T, this is equivalent to Y(t)=(D∧t,D∧T∧t), where x∧y=min(x,y).

In a general setting, consider a user-specified “win indicator” function 𝒲 such that

$$𝒲\left(Y_{i},Y_{j}\right)\left(t\right)=I\left\{Y_{i}\left(t\right)\;\text{wins}\;\text{compared}\;\text{to}\;Y_{j}\left(t\right)\right\},$$

where $Y_{i}(t)$ and $Y_{j}(t)$ represent the outcomes data for two distinct subjects. (Subscript will be used to denote subject affiliation without further clarification.) Likewise, $𝒲\left(Y_{j},Y_{i}\right)(t)=1$ means that subject i “loses” to subject j by time t. A “tie” arises when $𝒲\left(Y_{i},Y_{j}\right)(t)=𝒲\left(Y_{j},Y_{i}\right)(t)=0$. The index t plays an important role here, as the win-loss-tie status changes over time depending on the events available in the specified window.

The function 𝒲 is often specified to prioritize death over nonfatal events. For example, if T denotes time to the first nonfatal event, a common two-tiered comparison is given by

$$𝒲\left(Y_{i},Y_{j}\right)(t)=\underset{\text{Win}\;\text{on}\;\text{survival}}{\underbrace{I\left(D_{j}<D_{i}\wedge t\right)}}\;+\;\underset{\text{Tie}\;\text{on}\;\text{survival,}\;\text{win}\;\text{on}\;\text{nonfatal}\;\text{event}\;}{\underbrace{I\left(D_{i}\wedge D_{j}>t,\;T_{j}<T_{i}\wedge t\right)}}.$$

Alternatively, the nonfatal component can be compared through the cumulative frequency rather than the first occurrence. This involves replacing $T_{j}<T_{i}\wedge t$ with $N_{i}(t)<N_{j}(t)$, and possibly adding comparisons on event times to break equal-frequency ties [13].

Let z denote a p-dimensional vector of covariates. To evaluate their effects on the (prioritized) composite outcomes through the win ratio, the proportional win-fractions (PW) [5] specifies that

(1) $$\frac{pr\left\{𝒲\left(Y_{i},\;Y_{j}\right)(t)\;=\;1\;|\;z_{i},\;z_{j}\right\}}{pr\left\{𝒲\left(Y_{j},\;Y_{i}\right)(t)\;=\;1\;|\;z_{j},\;z_{i}\right\}}=\exp\left\{\beta^{T}\left(z_{i}-z_{j}\right)\right\},$$

where β is a p-dimensional vector of regression coefficients, each representing the log-win ratio associated with one unit increase in the corresponding covariate (holding others constant). The proportionality assumption on the win-loss fractions ensures that the win ratios are constant over time (similar to the role the proportional hazards assumption plays in the Cox model).

With a sensible 𝒲 that prioritizes death over nonfatal events, our earlier work has shown how to estimate β using censored data [5]. Let C denote the (independent) censoring time. A random n-sample of observed data then consist of

(2) $$\left\{Y_{i}\left(X_{i}\right),\;X_{i},\;z_{i}\right\}\;(i=1,\;\ldots \;,\;n),$$

where $X_{i}=D_{i}\wedge C_{i}$ is the observation time for the ith subject.

For a pair of indices 1≤i≠j≤n, write $\delta_{ij}=𝒲\left(Y_{i},Y_{j}\right)\left(X_{i}\wedge X_{j}\right)$, the win indicator for subject i over j during their *shared* observation window. Also, write $z_{ij}=z_{i}-z_{j}$, the vector of covariate differences. Consider the set of all “comparable” pairs

(3) $$ℛ=\left\{(i,\;j)\;:\;\delta_{ij}+\delta_{ji}\neq 0,\;0\leq i<j\leq n\right\}.$$

Using this set of notation, we can, up to a scaling factor, rewrite the U-estimating function from Section 3.2 of Mao and Wang (2021) [5] (with the recommended time-constant weight) as

(4) $$U_{n}(\beta )={\left|ℛ\right|}^{-1}\sum_{(i,j)\in ℛ}z_{ij}\left\{\delta_{ij}-\frac{exp\left(\beta^{T}z_{ij}\right)}{1\;+\;exp\left(\beta^{T}z_{ij}\right)}\right\},$$

where |ℛ| is the cardinality of (i.e., total number of pairs in) ℛ. An unregularized estimator of β is thus obtained by solving $U_{n}(\hat{\beta })=0$, through the Newton-Raphson algorithm.

### 2.2 Regularization and computation

From a numerical perspective, (4) is indistinguishable from the score function of a logistic regression model, albeit without an intercept, for binary response $\delta_{ij}$ against covariates $z_{ij}$ from a “random” sample of size |ℛ|. In fact, if we momentarily ignore the correlations between the pairs, we can even interpret the win-loss modeling as a kind of “conditional” logistic regression commonly used in matched case-control designs, where a case (winner) is always paired with a control (loser). This connection between win ratio and logistic regression can be exploited in regularizing β.

We adopt the elastic net approach [10]. To penalize the $L_{1}/L_{2}$ norms of β, consider the objective function

(5) $$\begin{array}{c} l_{n}(\beta ;\lambda )=-|ℛ{|}^{-1}\sum_{(i,j)\in ℛ}\left[\delta_{ij}\beta^{T}z_{ij}-log\left\{1+exp\left(\beta^{T}z_{ij}\right)\right\}\right]\\ +\;\lambda \{(1-\alpha )\|\beta {\|}_{2}^{2}/2+\alpha \|\beta {\|}_{1}\}, \end{array}$$

where λ≥0 controls the degree of regularization and the mixture parameter α∈[0,1] controls the balance between lasso and ridge regression. Straightforwardly, the standard estimator that solves $U_{n}(\hat{\beta })=0$ corresponds to $\hat{\beta }=arg\;{min}_{\beta }\;l_{n}(\beta ;\;0)$.

Unsurprisingly, (5) takes the same form as the penalized negative log-likelihood of a (no-intercept) logistic regression on the pairs (i,j)∈ℛ. Hence, it can be minimized using existing software packages designed for such tasks. For example, the pathwise solution

(6) $$\hat{\beta }(\lambda )=arg\;\underset{\beta }{min\;}\;l_{n}(\beta ;\;\lambda )$$

can be efficiently computed by glmnet::glmnet(x, y, family = “binomial”, intercept = FALSE, lambda), where x is the “covariate matrix” containing the $z_{ij}$, y is the “response vector” of the $\delta_{ij}$, intercept = FALSE removes the intercept, and lambda provides the optional user-specified λ vector (sensible values are generated by default) [11, 14].

#### Remark 1.

The no-intercept constraint is essential in our problem. By definition, all pairs (i,j)∈ℛ contain a win and a loss, and whether $\delta_{ij}=1$ (win) or 0 (loss) depends on the arbitrary ordering of i and j (in (3), we specified i<j. This means that a symmetry must exist between the two indices. A non-zero intercept would violate this symmetry and make the result dependent on the nominal “prevalence” of wins against losses, an artifact of subject ordering.

### 2.3 Proper cross-validation via partitioning of subjects, not pairs

In practice, the value of λ is tuned for optimal performance. This is commonly done through cross-validation, where the training data (2) are partitioned into multiple subsets (or folds). The model is fitted on a subset of the data (analysis set) and evaluated on the remaining subset (validation set). This process is repeated across all folds, and the average performance metric is computed for each candidate value of λ. The λ value that maximizes the performance metric is selected as the optimal.

Since we have recast our problem as a regularized logistic regression, it seems intuitive to tune model parameters as such. This would involve randomly partitioning the pairs in ℛ into different folds and utilizing glmnet’s built-in routines, such as cv.glmnet(), to perform cross-validation based on, say, classification accuracy.

This, however, would be a mistake. To start, notice that ℛ has a quadratically inflated size compared to the original sample due to pairing, i.e., $|ℛ|=O\left(n^{2}\right)$. Treating the pairs as if they were independent data would be too optimistic about the information they contain. Additionally, since each subject appears in many pairs, the analysis and validation sets remain correlated at the subject level even when they are separated at the pair level. Both factors contribute to overfitting (to get a flavor, refer to Fig. 2 for the HF-ACTION study).

Valid tuning requires us to split the n-sample (2) at the subject level. Let ${𝒮}^{\left(1\right)},{𝒮}^{\left(2\right)},\ldots ,{𝒮}^{\left(K\right)}$ be a random partition of the index set {1,…,n}. To balance the outcomes across the folds, randomization can be stratified on the outcome status. To evaluate performance on ${𝒮}^{\left(k\right)}$, the model will be fitted on ${𝒮}^{\left(-k\right)}=\{1,\;\ldots \;,\;n\}\backslash {𝒮}^{\left(k\right)}$. This ensures that each subject’s data are contained entirely within either the analysis or validation set.

More specifically, write

$${ℛ}^{\left(-k\right)}=\{(i,\;j):\delta_{ij}+\delta_{ji}\neq 0;\;i<j;\;i,\;j\in {𝒮}^{\left(-k\right)}\},$$

the set of comparable pairs in the analysis set. Then, following the previous section, we fit a pathwise model on ${𝒮}^{\left(-k\right)}$ by solving

$${\hat{\beta }}^{\left(-k\right)}\left(\lambda \right)=\arg\;min\;l_{n}^{\left(-k\right)}\left(\beta ;\;\lambda \right),$$

where $l_{n}^{\left(-k\right)}(\beta ;\;\lambda )$ is defined identically to $l_{n}(\beta ;\;\lambda )$ except that ℛ is replaced by ${ℛ}^{\left(-k\right)}$.

Suppose that the predictive performance of the fitted PW model (1) with parameter optimal performance, we would choose theb that maximizes the average measure ${\hat{\beta }}^{\left(-k\right)}(\lambda )$ is measured on ${𝒮}^{\left(k\right)}$ by some metric ${𝒞}^{\left(k\right)}(\lambda )$, for each value of λ. Then for across the folds, i.e., $\lambda_{\text{opt}\;}=arg\;{max}_{\lambda }\;𝒞(\lambda )$, where $𝒞(\lambda )=K^{-1}\sum_{k=1}^{K}{𝒞}^{\left(k\right)}(\lambda )$.

### 2.4 Generalized concordance index to measure predictive performance

A key question is, how do we define ${𝒞}^{\left(k\right)}(\lambda )$? Since predicting win-loss is fundamentally a classification problem, classification accuracy among the comparable pairs in the validation set becomes a natural target.

Write ${ℛ}^{\left(k\right)}=\{(i,\;j)\;:\;\delta_{ij}+\delta_{ji}\neq 0;\;i<j;\;i,\;j\in {𝒮}^{\left(k\right)}\}$, the set of comparable pairs in the validation set. Under model (1), the probability of a win for subject i against j given their comparability is

$$\mu \left(z_{i},\;z_{j};\;\beta \right)=\frac{exp\{\beta^{T}\left(z_{i}\;-\;z_{j}\right)\}}{1\;+\;exp\{\beta^{T}\left(z_{i}\;-\;z_{j}\right)\}}$$

(which is also clear from (4)), and the probability of vice versa is $1-\mu \left(z_{i},\;z_{j};\;\beta \right)$. By symmetry, we classify a win-loss pair correctly if $\mu \left(z_{i},\;z_{j};\;\beta \right)>0.5$ when i wins and $\mu \left(z_{i},\;z_{j};\;\beta \right)<0.5$ when j wins.

This approach also aligns well with Harrell’s C(oncordance)-index, commonly used in regression modeling of censored time-to-event data [15, 16]. Harrell’s C evaluates the proportion of all comparable (or “usable”) pairs where the predicted risk score (e.g., linear predictor in the Cox model) correctly ranks the subjects in accordance with their observed survival outcomes. The idea has since been extended to hierarchical endpoints [17].

In our case, we consider $\beta^{T}z_{i}$ “win score” for subject i. Clearly, the following equivalence holds:

$$\mu \left(z_{i},\;z_{j};\;\beta \right)>0.5\Leftrightarrow \beta^{T}z_{i}>\beta^{T}z_{j};$$

$$\mu \left(z_{i},\;z_{j};\;\beta \right)=0.5\Leftrightarrow \beta^{T}z_{i}=\beta^{T}z_{j};$$

$$\mu \left(z_{i},\;z_{j};\;\beta \right)<0.5\Leftrightarrow \beta^{T}z_{i}<\beta^{T}z_{j}.$$

Though not meaningful in absolute terms, the win score can be thought of as a relative measure of the propensity to win (on the hierarchical scale of the outcomes). With ${𝒮}^{*}$ denoting a generic index set, and ${ℛ}^{*}=\{(i,\;j)\;:\;\delta_{ij}+\delta_{ji}\neq 0;\;i<j;\;i,\;j\in {𝒮}^{*}\}$ the set of comparable pairs within, we can succinctly express the classification accuracy, or equivalently the C-index of the predicted win score, as

(7) $$𝒞({𝒮}^{*};\beta )={\left|{ℛ}^{*}\right|}^{-1}\sum_{(i,j)\in {ℛ}^{*}}\left[I\left\{\left(2\delta_{ij}-1\right)\left(\beta^{T}z_{i}-\beta^{T}z_{j}\right)>0\right\}+2^{-1}I\left(\beta^{T}z_{i}=\beta^{T}z_{j}\right)\right],$$

where the second term on the right hand side adjusts for (predicted) ties. Applying (7) to the evaluation of ${\hat{\beta }}^{\left(-k\right)}(\lambda )$ on ${𝒮}^{\left(k\right)}$, we have that

$${𝒞}^{\left(k\right)}\left(\lambda \right)=𝒞\left({𝒮}^{\left(k\right)};{\hat{\beta }}^{\left(-k\right)}\left(\lambda \right)\right).$$

### 2.5 Variable importance and risk prediction

After determining $\lambda_{\text{opt}}$ through cross-validation, we refit the model using the entire training data (2) to obtain ${\hat{\beta }}_{opt}=\hat{\beta }\left(\lambda_{opt}\right)$. If α>0 in (5) so that there is a lasso element, some components of ${\hat{\beta }}_{opt}$ will be exactly zero, indicating the corresponding covariates have been excluded from the model.

Among the selected covariates, we can measure their relative importance by the magnitude of the fitted regression coefficients when the covariates are standardized [18, 19]. Standardization puts all covariates on the same scale, allowing for a direct comparison. Larger magnitudes indicate stronger associations with the win-loss outcomes. Alternatively, some authors have considered the frequency with which a covariate is selected as λ varies, which reflects the variable’s stability and robustness in the selection process [20, 21].

Typically before analysis, a portion of the data is set aside to test the performance of the final model. Denote this test set data as

(8) $$\{Y_{i}^{*}(X_{i}^{*}),\;X_{i}^{*},\;z_{i}^{*}\}\;\left(i=1,\;\ldots \;,\;m\right).$$

For the ith subject in (8), a predicted win score is calculated by ${\overset{ˆ}{w}}_{i}^{*}={\hat{\beta }}_{opt}^{T}z_{i}^{*}$, or equivalently, a “risk” score by ${\overset{ˆ}{r}}_{i}^{*}=-{\overset{ˆ}{w}}_{i}^{*}$. With ${ℛ}^{*}=\{(i,j):\delta_{ij}^{*}+\delta_{ji}^{*}\neq 0,\;0\leq \;i<j\leq m\}$ denoting the set of comparable pairs, we can measure the overall model performance by

(9) $${𝒞}^{*}={\left|{ℛ}^{*}\right|}^{-1}\sum_{(i,j)\in {ℛ}^{*}}[I\{(2\delta_{ij}^{*}-1)({\overset{ˆ}{w}}_{i}^{*}-{\overset{ˆ}{w}}_{j}^{*})>0\}+2^{-1}I({\overset{ˆ}{w}}_{i}^{*}={\overset{ˆ}{w}}_{j}^{*})],$$

following (7).

As a unique feature of hierarchical composite endpoints, a component-wise C-index can be calculated to assess the prognostic value for a specific component. This is achieved by redefining $\delta_{ij}^{*}$ and ${ℛ}^{*}$ in (9) to focus exclusively on wins and losses determined by either death or nonfatal events, for example.

Unlike the Cox model, the PW model lacks a “baseline” function that, when combined with the risk score, allows for the prediction of event probabilities. This limitation, however, can be addressed through a nonparametric approach to risk stratification. Specifically, the risk score spectrum can be divided into L distinct strata based on appropriate quantiles from the training data. Within each stratum, Kaplan-Meier curves can be constructed for overall survival and event-free survival. Collectively, these curves provide probabilities for a hierarchical sequence of states, such as being event-free, alive but having experienced a nonfatal event, and dead, at any given time. For a subject in the test set, these probabilities can be predicted by identifying the risk stratum corresponding to their predicted score and applying the stratum-specific prognosis.

### 2.6 Software implementation

The proposed procedures are implemented in the R-package wrnet, available on GitHub at https://lmaowisc.github.io/wrnet. Key functionalities include wrnet() for pathwise solution in (6), cv_wrnet() for K-fold cross-validation, and test_wrnet() for evaluation of test performance. Additionally, the package provides various helper functions to calculate or visualize C-indices, variable importance, and more.

The main steps in data analysis are outlined below, along with instructions for executing them using wrnet functions.

#### 1. Data preparation

- Initial data frame df in long format with id, time, status columns: subject identifiers, event times, and event status (1 for death; 2 for nonfatal event; 0 for censoring), along with covariate columns.
- Partition into training and test sets by a specified proportion:

wr_split(df, prop = 0.8)
  - Returns a list of training data df_train and test data df_test;
  - Default split stratified by outcome status.

#### 2. Cross-validation

- Perform *k*-fold cross-validation on df_train:

cv_wrnet(id, time, status, Z, k = 10, ...)
  - Returns a tibble with columns lambda and concordance;
  - Identify optimal lambda_opt maximizing concordance;
  - Additional arguments, such as α∈[0,1] (default is 1), passed to glmnet() for pairwise logistic regression on each fold.

#### 3. Fit final model

- Final fit on df_train: under optimal lambda_opt:

final_fit <- wrnet(id, time, status, Z, lambda = lambda_opt, ...)
  - $\hat{\beta }\left(\lambda_{opt}\right)$: final_fit$beta;
  - Variable importance: vi_wrnet(final fit);
  - Additional arguments passed to glmnet().

#### 4. Test performance

- Calculate overall and component-wise C-indices on df_test:

test_wrnet(final_fit, df_test)

To illustrate the process, here is a simple code example analyzing the German breast cancer study data contained in the WR package. It takes only a few seconds to run and can be reproduced by the user.


library(tidyverse) # For data wrangling and visualization 
library(WR) # To get the gbc data for illustration
source(“R code/basic_functions.R”)
# Load data 
data(“gbc”)
df <- gbc # n = 686 subjects, p = 9 covariates 
df
#>   id    time   status hormone age menopause size grade ...
#>1   1  43.836066     2       1  38         1   18     3
#>2   1  74.819672     0       1  38         1   18     3 
#>3   2  46.557377     2       1  52         1   20     1
#>4   2  65.770492     0       1  52         1   20     1
#>5   3  41.934426     2       1  47         1   30     2
#>...
# Data partitioning -----------------------------------set.seed(123) 
obj_split <- df |> wr_split() 
# Take training and test set 
df_train <- obj_split$df_train 
df_test <- obj_split$df_test
# 10-fold CV ------------------------------------------
set.seed(1234)
obj_cv <- cv_wrnet(df_train$id, df_train$time, df_train$status,
                       df_train |> select(-c(id, time, status))) 
# Plot CV results (C-index vs log-lambda) 
obj_cv |> 
  ggplot(
    aes(x = log(lambda), y = concordance)
  ) +
  geom_point() + 
  geom_line() 
# Optimal lambda
lambda_opt <- obj_cv$lambda[which.max(obj_cv$concordance)]
# Final model -----------------------------------------
final_fit <- wrnet(df_train$id, df_train$time, df_train$status,
                       df_train |> select(-c(id, time, status)),
		       lambda = lambda_opt) 
# Estimated coefficients
final_fit$beta
#> 8 × 1 sparse Matrix of class “dgCMatrix”
#>                            s0
#> hormone            0.306026364
#> age                0.003111462
#> menopause          .
#> size       −0.007720497
#> grade      −0.285511701
#> nodes      −0.082227827
#> prog_recp          0.001861367
#> estrg_recp         . 
# Variable importance plot 
final_fit |>
  vi_wrnet() |> 
  vip()
# Test model performance ------------------------------
test_result <- final_fit |> test_wrnet(df_test) 
# Overall and event-specific C-indices
test_result$concordance
#> # A tibble: 3 × 2
#> component concordance
#> <chr> <dbl>
#> 1 death         0.724
#> 2 nonfatal      0.607
#> 3 overall       0.664


## 3 Simulation studies

Simulations were conducted to assess the performance of the proposed regularized win ratio regression (wrnet) in feature selection and risk prediction. We focused on the comparison with regularized Cox model [12] for time to the first event, the standard approach to composite outcomes.

Consider a p-vector of covariates $z=\left(z_{\cdot 1},\;z_{\cdot 2},\;\ldots ,\;z_{\cdot p}\right)$, where each component $z_{\cdot k}\;\sim \;N(0,\;1)$. For a realistic setting, we assume a first-order auto-regressive correlation structure such that the correlation between two adjacent components is ρ=0.1. Given z, we generated the bivariate outcomes (*D,T*) through the joint model:

(10) $$pr(D>s,T>t|z)=exp\left(-{\left[{\left\{exp\left(-\beta_{D}^{T}z\right)\lambda_{D}s\right\}}^{\kappa }+{\left\{exp\left(-\beta_{H}^{T}z\right)\lambda_{H}t\right\}}^{\kappa }\right]}^{1/\kappa }\right),$$

where the effects of z on D (survival time) and T (nonfatal event time) are reflected through $\beta_{D}$ and $\beta_{H}$, respectively. In particular, $exp\left(-\beta_{D}\right)$ and $exp\left(-\beta_{H}\right)$ contain the hazard ratios on the marginal distributions of the two components. We set $\lambda_{D}=0.1,\;\lambda_{H}=1$, and κ=1.25. The last parameter induces a Kendall’s rank correlation of $1-\kappa^{-1}=20\%$ between the components [22].

For censoring, let C~Un[0.2,4]∧Expn(0.02), mimicking the earlier of a uniformly distributed administrative censoring point and an exponentially distributed loss to follow-up point. At baseline (z=0), this set-up gives rise to a death rate of about 20%, a nonfatal event rate of about 75%, and a first composite event rate of about 80%.

Because model performance will likely depend on the patterns of covariate effects on the two outcome components, we considered two scenarios:

- Scenario 1 (same effect pattern):
$$\beta_{D}=\beta_{H}=(\underset{10}{\underbrace{0.5,\;\ldots \;,\;0.5}},\;\underset{p-10}{\underbrace{0,\;\;\ldots \;,\;0}}).$$
- Scenario 2 (different effect patterns):
$$\beta_{D}=(\underset{5}{\underbrace{0.75,\;\ldots \;,\;0.75}},\;\underset{p-5}{\underbrace{0,\;\ldots \;,\;0}});$$
$$\beta_{H}=(\underset{5}{\underbrace{0,\;\ldots \;,\;0}},\;\underset{5}{\underbrace{0.75,\;\ldots \;,\;0.75}},\;\underset{p-10}{\underbrace{0,\;\ldots \;,\;0}})$$

Hence, while the first ten covariates influence both components in scenario 1, the first five influence death and the sixth to tenth influence the nonfatal event in scenario 2. We set p=20.

For each sample size n=200,500,1000 and 2000, we generated N=1000 datasets. For each dataset, we followed the workflow described in the previous section to train the optimal wrnet model on 80% of the data (including a 10-fold cross-validation) and evaluate its performance on the remain 20% test set. As comparison, a similar set of procedures were performed for the regularized Cox model for time to the first event using glmnet(x, y, family = “cox”). For both models, the mixture parameter is fixed at α=1, yielding lasso-type models.

First, consider the accuracy of variable selection. In both scenarios, the first ten covariates have a direct effect on the outcomes, while the last ten do not. We thus assess the sensitivity (i.e., frequency of being selected) for the first set and specificity (i.e., frequency of being dropped) for the second set. The results are summarized in Table 1.

While both methods have high sensitivities in identifying all important covariates in the first scenario, the Cox model missed substantial proportions of the first five covariates in the second scenario (especially for the smaller sample sizes). This, however, is not surprising. Recall that these covariates in scenario 2 act on the composite outcomes only through death. Since death constitutes only a small fraction of the first composite event (as is common in many applications [23, 24]), their effects become harder to detect if death is not prioritized.

For specificity, the performance of the two methods remains similar in the first scenario but appears to diverge with the sample size in the second – the win ratio’s performance improves with larger samples, while the Cox model’s deteriorates. A possible explanation is that, due to the lack of prioritization, covariate effects tend to be more diffuse on the first event. As many covariates influence the first event indirectly through their correlations with direct predictors of nonfatal events, this causes Cox model to be more liberal in selecting variables. Notably, aside from a slight disadvantage in specificity in scenario 2 with n=200, the win ratio consistently outperforms the Cox model on both metrics.

**Fig. 1:**
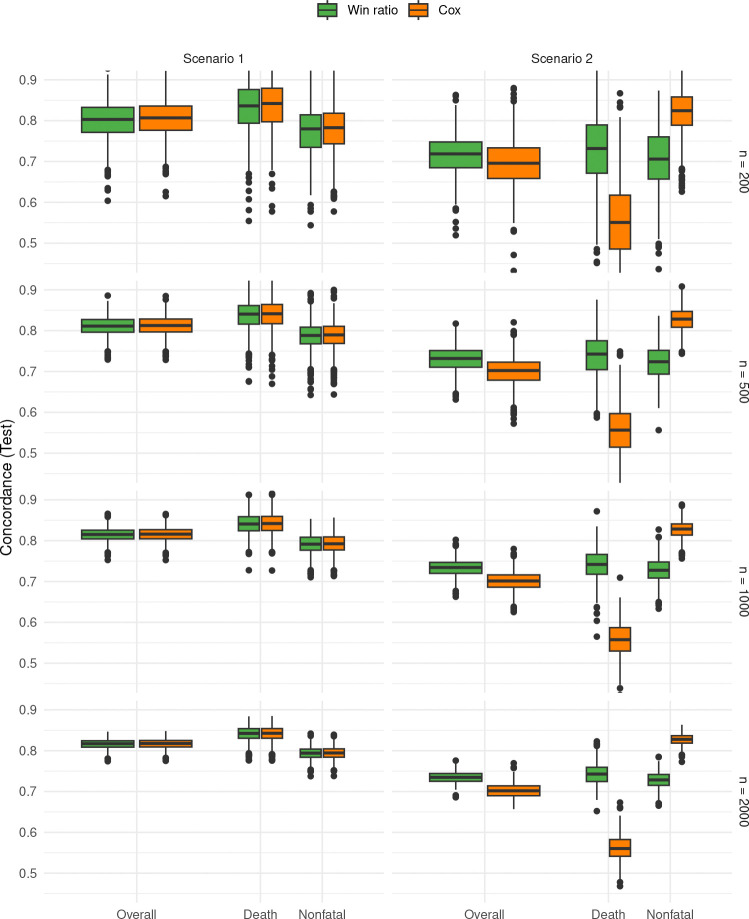
Regularized win ratio (wrnet) and Cox models trained on 80% of data at α=1 (lasso) and tested on remaining 20%.

Next, we examine the prediction accuracy of the trained models. The distributions of test C-indices by both models across the scenarios and sample sizes are plotted in Fig. 1. In the first scenario, the predictive performance of the two methods on the composite outcomes, both overall and component-wise, appears so similar that they are virtually indistinguishable. In the second scenario, however, the Cox model performs exceedingly poorly on the prognosis of death (for reasons mentioned earlier), leading to a considerable disadvantage in overall concordance, despite its stronger performance on the nonfatal component.

Different simulation settings, such as scenarios with a mixture α=0.5, higher death rates, or a higher-dimensional z, are explored in the supplementary material available online. These simulations have demonstrated the robustness of the regularized win ratio approach, which consistently outperforms the Cox model for time to the first event, particularly when covariates have differing effects on the outcome components. Notably, this advantage does not depend on whether the covariate effects are stronger on death or nonfatal events. Instead, it stems from the win ratio’s inherent prioritization scheme, which is consistent with the interpretation, and thus evaluation, of the different outcomes.

## 4 A real-world application

The HF-ACTION study (Heart Failure: A Controlled Trial Investigating Outcomes of Exercise Training) was a multicenter randomized clinical trial designed to evaluate the effects of aerobic exercise training in patients with heart failure and reduced ejection fraction (HFrEF) [6]. The study enrolled over 2,000 participants who were randomly assigned to either a supervised exercise training program or usual care. The primary outcome was time to the first occurrence of all-cause death or hospitalization, while secondary outcomes assessed health-related quality of life and functional capacity.

Although the primary analysis did not find a statistically significant effect for the treatment, subsequent ones have demonstrated the prognostic value of various clinical and functional measures, such as baseline cardiopulmonary exercise (CPX) parameters [6, 25, 26]. In addition, the study has documented extensive information on patient demographics, biomarkers, comorbidities, medical history, and functional assessments, providing a comprehensive dataset for exploring risk factors in heart failure. Along with its hierarchical outcomes (mortality > hospitalization), the study presents an ideal example for applying and evaluating our regularized win ratio regression (wrnet) approach.

To do so, consider high-risk subcohort consisting of 426 patients with baseline CPX duration of nine minutes or less. Over the median follow-up of 2.3 years, 93 (22%) patients died, 315 (73.9%) were hospitalized at least once, and 326 (76.5%) experienced a composite event. There are a total of p=153 candidate features to predict the outcomes, including the treatment indicator.

**Fig. 2:**
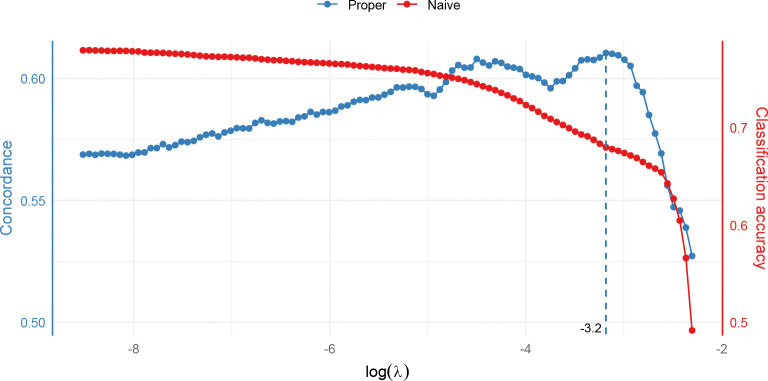
Proper versus naive cross-validation in HF-ACTION study. The naive approach is biased toward overfitting due to inflated sample size and overlap between analysis and validation sets.

To build a robust and parsimonious model, we first split the data using a 4:1 ratio into a training set (N=341) and a test set (N=85). The split is stratified by the outcome status to ensure balanced representation of outcomes in both sets. Then, setting α=1 for a lasso-type model, we perform a 10-fold cross-validation using cv_wrnet() to find the optimal λ. The relationship between the validation C-index and log(λ) is plotted in Fig. 2. The C-index is maximized at $log\left(\lambda_{opt}\right)=-3.2$. For comparison, we also overlay the cross-validation curve generated by using cv.glmnet() on the pooled pairwise data. Not only does this approach lead to an overly optimistic assessment of classification accuracy (note the different y-axes), it is also severely biased toward overfitting, with the performance measure continuing to rise as log(λ) decreases beyond its lower limit (≈−8.6). Such overfitting would significantly curtail model generalizability if the optimal λ were determined in this way.

Given the optimal λ, we build our final win ratio model, which includes 20 out of the 159 features. To assess model performance in proper context, a regularized Cox model for time to the first event is also fitted on the same training data. The overall and component-wise C-indices of the two models on the test set are presented in Table 2. The win ratio outperforms the Cox model in the overall C-index, achieving 0.605 compared to 0.572 – a considerable margin given the moderate predictive strengths of both models. This difference is primarily driven by death, where the win ratio shows an advantage of 5.5 percentage points, although a smaller lead also exists for hospitalization.

**Fig. 3:**
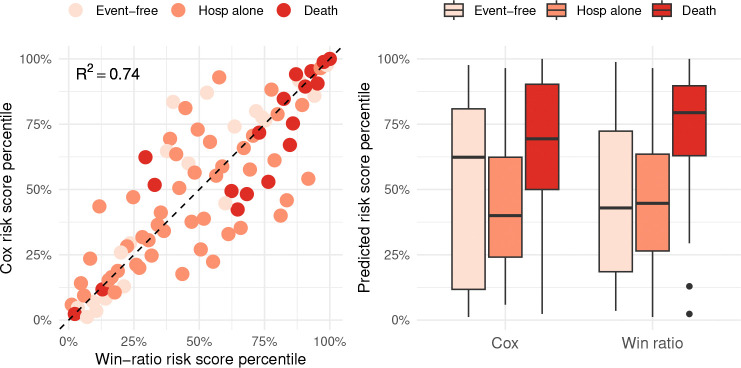
Risk scores predicted by regularized win ratio versus Cox models and their relationships with patient outcomes in the test set.

To illustrate how the predicted risk scores correlate with each other and with patient outcomes in the test set, the left panel of Fig. 3 shows a scatter plot of the (rank-normalized) scores from both models, stratified by outcome status. To interpret the graph, note that points below the unit-sloped reference line receive a lower risk-score percentile from the Cox model than from the win ratio, and that points above do vice versa. Despite the substantial correlation $\left(R^{2}=0.74\right)$, the Cox model under-predicts the risk for more patients who died than it over-predicts, highlighting its limitation due to the lack of prioritization. This limitation is also evident in the right panel, where the risk scores are grouped by outcome status. While the score distributions for patients who were hospitalized but remained alive are comparable across the models, the win ratio tends to generate higher and more concentrated scores for those who died, resulting in more accurate prognosis both component-wise and overall.

**Fig. 4:**
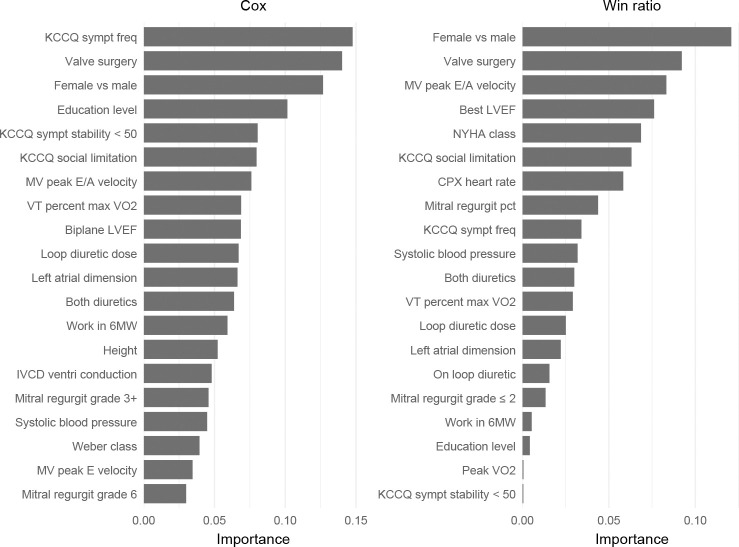
Top 20 variables selected by regularized Cox and win ratio regression models, along with their importance as measured by the magnitude of the coefficients for standardized variables.

The Cox model selects 32 features, compared to 20 by the win ratio. The top 20 most important variables identified by each model, along with their quantified importance, are shown in Fig. 4. There is considerable overlap between the two sets – 13 variables are on both lists. These include patient sex (female vs male), history of valve surgery (y vs n), MV peak E/A velocity, KCCQ (Kansas City Cardiomyopathy Questionnaire) social limitation, systolic blood pressure, on both loop and non-loop diuretics (y vs n), VT (ventilatory threshold) percent max VO2, loop diuretic dose, left atrial dimension, work in six-minute walk (6MW), education level (1–6), and KCCQ symptom stability < 50 (y vs n).

Finally, we refit the win ratio model on the entire dataset (combining training and test sets) using the 20 selected covariates. The results are summarized in Table 3. As the top predictor selected by the regularized model, patient sex is a strong determinant of outcomes, with females 89% more likely to fare better than males. Other significant predictors include a history of valve surgery, work done during the baseline six-minute walk, and so on. Of note, VT percent max VO2 (%), defined as the ratio of VO2 at ventilatory threshold to peak VO2, appears to have a negative impact on patient outcomes, which is likely attributable to its inverse association with peak VO2, a key marker of aerobic capacity. This inverse relationship may have also diminished the positive effects commonly associated with higher peak VO2 as an independent predictor [27]. Further refinements to the model may be necessary to enhance interpretability while maintaining its predictive performance.

## 5 Discussion

We have developed an elastic net-type approach to regularize win ratio regression for variable selection and risk prediction. The prioritization scheme of the win ratio ensures that both variable selection and risk prediction are guided by critical events such as death. This addresses a key limitation of traditional time-to-first-event analysis, which tends to overemphasize secondary events due to their earlier and more frequent occurrences.

Indeed, as demonstrated by simulations and an application to the HF-ACTION study, regularized win ratio models offer solutions that are better aligned with the real-world impact of patient outcomes compared to regularized Cox models, which focus exclusively and indiscriminately on the first event. The accompanying wrnet package combines efficient computational routines from the glmnet package with a user-friendly interface, streamlining model fitting, tuning, and testing. This tool can be routinely utilized by practitioners to address challenges in variable selection and risk prediction for hierarchical composite endpoints, enhancing both the interpretability and generalizability of their models.

In numerical studies, we have focused on the standard, two-tiered comparison with death and a nonfatal event. As the general form of the PW model (1) suggests, however, a customized win function can be specified to make more efficient use of the outcomes data, such as recurrent events (these features will be incorporated in wrnet in future updates). Additionally, further simulations and applications are needed to explore model performance under varying α, or even to investigate setting α as a tuning parameter [28].

For risk prediction we have proposed to use the (regularized) linear predictor from the PW model as the win score, or equivalently, its negative as the risk score. This risk score has been compared with the linear predictor from the Cox model, a commonly used risk score for univariate survival data [16, 29], in the prognosis of hierarchical outcomes. Interestingly, the two risk scores have a deeper connection – they are completely equivalent in the univariate case and coincide with each other in limited scenarios of hierarchical endpoints where covariate effects are the same across components [5, 30]. The appeal of the PW risk score is two-fold. First, it assigns a numeric value to each *subject* despite the pairwise specification of the model. Second, both its estimation and regularization are driven by the hierarchical nature of the outcomes. It is thus unsurprising that the PW score generally aligns better with the outcomes than does the Cox score for the first event.

In evaluating model performance, we have considered a straightforward extension of Harrell’s C-index, adjusted to account for the hierarchical outcomes. However, like the original Harrell’s C, this metric is influenced by the censoring distribution in the test set. To explicitly account for the effects of follow-up time, Uno et al. (2011) [31] defined a time-dependent C-index as a function of the observation window and proposed an inverse probability censoring weighting (IPCW) technique for its estimation. Cheung et al. (2019) [17] extended this approach to hierarchical outcomes. In the context of win ratio models, a time-dependent C-index could provide valuable insights into how the accuracy of risk prognosis evolves over time. This concept may also be applied to building stronger models by targeting time windows during tuning where the predictive power of the features is maximized.

The proposed framework has certain limitations. First, the computational cost associated with pairwise comparisons may become prohibitive for very large sample sizes. At present, the quadratic growth of computational burden seems inevitable given the intransitivity of the win ratio [32], which prevents inferring all pairwise results based on a smaller set of comparisons. However, future work could explore more efficient algorithms or approximate methods to scale the approach. For example, it might not be necessary to use all pairwise data in tuning the penalty parameter. Additionally, potential violations of the proportionality assumption on the win-loss probabilities can take a toll on model performance. Finding a way to accommodate time-varying effects would enhance model robustness.

Most importantly, the inability of a regression model to flexibly handle nonlinear effects and interactions may be the strongest bottleneck limiting model performance. While the use of a linear predictor simplifies interpretation, it may miss out on more complex patterns. For example, certain biomarkers may only have prognostic value above or below a certain threshold, or their effects may depend on the levels of other covariates. In the univariate case, survival trees or random forests have shown great promise in addressing such complexities and thereby achieving better results [33, 34]. Extending these approaches to hierarchical endpoints would be a suitable next step.

## Figures and Tables

**Table 1:** Sensitivity and specificity of variable selection by regularized win ratio (Cox) regression models (α=1) in different scenarios.

	*n* = 200		*n* = 500		*n* = 1000		*n* = 2000	
	S1	S2	S1	S2	S1	S2	S1	S2

Sensitivity								
*z*·1	1 (1)	0.98 (0.60)	1 (1)	1 (0.78)	1 (1)	1 (0.95)	1 (1)	1 (1)
*z*·2	1 (1)	0.97 (0.63)	1 (1)	1 (0.82)	1 (1)	1 (0.98)	1 (1)	1 (1)
*z*·3	1 (1)	0.98 (0.60)	1 (1)	1 (0.81)	1 (1)	1 (0.97)	1 (1)	1 (1)
*z*·4	1 (1)	0.98 (0.62)	1 (1)	1 (0.79)	1 (1)	1 (0.97)	1 (1)	1 (1)
*z*·5	1 (1)	0.98 (0.62)	1 (1)	1 (0.82)	1 (1)	1 (0.97)	1 (1)	1 (1)
*z*·6	1 (1)	0.97 (1)	1 (1)	1 (1)	1 (1)	1 (1)	1 (1)	1 (1)
*z*·7	1 (1)	0.98 (1)	1 (1)	1 (1)	1 (1)	1 (1)	1 (1)	1 (1)
*z*·8	1 (1)	0.98 (1)	1 (1)	1 (1)	1 (1)	1 (1)	1 (1)	1 (1)
*z*·9	1 (1)	0.97 (1)	1 (1)	1 (1)	1 (1)	1 (1)	1 (1)	1 (1)
*z*·10	1 (1)	0.97 (1)	1 (1)	1 (1)	1 (1)	1 (1)	1 (1)	1 (1)
Specificity								
*z*·11	0.55 (0.49)	0.52 (0.61)	0.62 (0.60)	0.54 (0.56)	0.70 (0.72)	0.64 (0.50)	0.76 (0.77)	0.71 (0.48)
*z*·12	0.55 (0.49)	0.55 (0.64)	0.62 (0.62)	0.56 (0.57)	0.71 (0.71)	0.62 (0.50)	0.79 (0.75)	0.81 (0.44)
*z*·13	0.53 (0.48)	0.54 (0.61)	0.63 (0.62)	0.53 (0.55)	0.71 (0.71)	0.62 (0.49)	0.76 (0.75)	0.79 (0.42)
*z*·14	0.53 (0.46)	0.53 (0.63)	0.63 (0.61)	0.52 (0.58)	0.72 (0.69)	0.62 (0.51)	0.77 (0.78)	0.75 (0.50)
*z*·15	0.54 (0.47)	0.56 (0.61)	0.61 (0.63)	0.55 (0.58)	0.73 (0.73)	0.61 (0.48)	0.78 (0.77)	0.77 (0.54)
*z*·16	0.55 (0.51)	0.59 (0.61)	0.62 (0.62)	0.59 (0.58)	0.73 (0.72)	0.63 (0.50)	0.77 (0.76)	0.69 (0.44)
*z*·17	0.56 (0.47)	0.55 (0.62)	0.63 (0.62)	0.56 (0.55)	0.70 (0.70)	0.62 (0.50)	0.78 (0.75)	0.73 (0.50)
*z*·18	0.53 (0.48)	0.55 (0.63)	0.63 (0.62)	0.57 (0.56)	0.72 (0.72)	0.60 (0.50)	0.78 (0.75)	0.73 (0.44)
*z*·19	0.55 (0.48)	0.53 (0.63)	0.61 (0.61)	0.56 (0.54)	0.72 (0.70)	0.64 (0.52)	0.76 (0.76)	0.73 (0.40)
*z*·20	0.56 (0.49)	0.52 (0.63)	0.63 (0.61)	0.59 (0.58)	0.71 (0.71)	0.62 (0.50)	0.76 (0.73)	0.77 (0.50)

S1, S2: Scenarios 1 and 2. Each entry is based on *N* = 1000 replicates.

**Table 2:** Test C-indices of regularized win ratio and Cox models (*α* = 1).

	Cox	Win ratio

Death	0.680	0.735
Hosp	0.522	0.543
Overall	0.572	0.605

**Table 3:** Final win ratio model refitted on entire data without regularization.

	Win ratio	95% CI	*p*-value

*Demographics*			
Female vs male	1.89	[1.42, 2.53]	<.001
Education level (1–6)	1.07	[0.98, 1.17]	0.153
*Medical History*			
Valve surgery (y vs n)	0.49	[0.29, 0.81]	0.005
On loop diuretic (y vs n)	0.89	[0.61, 1.30]	0.547
Loop diuretic dose (100mg)	0.91	[0.81, 1.01]	0.080
On both diuretics (y vs n)	0.69	[0.44, 1.10]	0.119
*Functional Measurements*			
KCCQ symptoms freq	1.01	[1.00, 1.01]	0.104
KCCQ social limitation score	1.00	[1.00, 1.01]	0.349
KCCQ symptom stability < 50 (y vs n)	0.75	[0.51, 1.11]	0.152
Work in 6MW (100kJ)	1.14	[1.00, 1.30]	0.045
NYHA class (III/IV vs II)	0.99	[0.74, 1.33]	0.964
Systolic blood pressure (mmHg)	1.01	[1.00, 1.01]	0.155
*CPX Parameters*			
VT percent max VO2 (%)	0.15	[0.03, 0.81]	0.028
CPX heart rate (bpm)	1.00	[0.99, 1.01]	0.540
Peak VO2 (mL/kg/min)	1.03	[0.98, 1.08]	0.269
*Echocardiographic or MR Measurements*			
MV peak E/A velocity (m/s)	0.85	[0.72, 1.01]	0.057
Best LVEF (%)	1.02	[1.00, 1.04]	0.066
Left atrial dimension (mm)	0.91	[0.77, 1.09]	0.318
Mitral regurgitation percent (%)	0.99	[0.98, 1.00]	0.019
Mitral regurgitation grade 2+ (y vs n)	1.35	[0.95, 1.91]	0.095

CI: Confidence interval.

## Data Availability

The proposed methodology is implemented in the R-package wrnet, openly available at https://lmaowisc.github.io/wrnet/. Data are available as per request to the NHLBI Data Repository (BioLINCC). Restrictions apply to the availability of these data.
